# Structural and Biochemical Investigation of Selected Pathogenic Mutants of the Human Dihydrolipoamide Dehydrogenase

**DOI:** 10.3390/ijms241310826

**Published:** 2023-06-28

**Authors:** Eszter Szabo, Eva Nemes-Nikodem, Krisztina Rubina Vass, Zsofia Zambo, Eszter Zrupko, Beata Torocsik, Oliver Ozohanics, Balint Nagy, Attila Ambrus

**Affiliations:** Department of Biochemistry, Institute of Biochemistry and Molecular Biology, Semmelweis University, 37-47 Tuzolto St., 1094 Budapest, Hungary

**Keywords:** lipoamide dehydrogenase, disease-causing mutation, X-ray crystallography, reactive oxygen species, alpha-keto acid dehydrogenase complexes

## Abstract

Clinically relevant disease-causing variants of the human dihydrolipoamide dehydrogenase (hLADH, hE3), a common component of the mitochondrial α-keto acid dehydrogenase complexes, were characterized using a multipronged approach to unravel the molecular pathomechanisms that underlie hLADH deficiency. The G101del and M326V substitutions both reduced the protein stability and triggered the disassembly of the functional/obligate hLADH homodimer and significant FAD losses, which altogether eventually manifested in a virtually undetectable catalytic activity in both cases. The I12T-hLADH variant proved also to be quite unstable, but managed to retain the dimeric enzyme form; the LADH activity, both in the *forward* and *reverse* catalytic directions and the affinity for the prosthetic group FAD were both significantly compromised. None of the above three variants lent themselves to an in-depth structural analysis via X-ray crystallography due to inherent protein instability. Crystal structures at 2.89 and 2.44 Å resolutions were determined for the I318T- and I358T-hLADH variants, respectively; structure analysis revealed minor conformational perturbations, which correlated well with the residual LADH activities, in both cases. For the dimer interface variants G426E-, I445M-, and R447G-hLADH, enzyme activities and FAD loss were determined and compared against the previously published structural data.

## 1. Introduction

**Figure 1:**
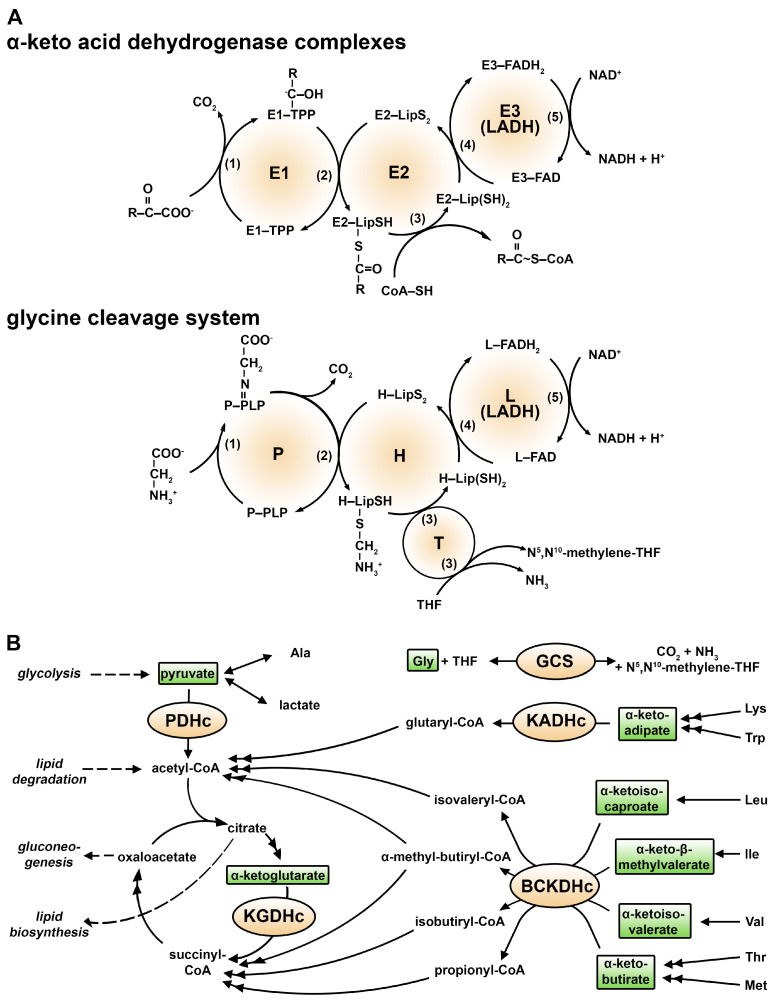
Reaction mechanisms and physiological functions of the hLADH-bearing multienzyme complexes. (**A**) Structural reaction schemes of the hLADH-bearing complexes. The α-keto acid dehydrogenase enzyme complexes and the glycine cleavage system (GCS) oxidatively decarboxylate their specific substrates in five consecutive steps (1–5) using several cofactors (TPP—thiamine pyrophosphate/diphosphate; LipS_2_/Lip(SH)_2_—oxidized/reduced lipoamide; FAD/FADH_2_—oxidized/fully reduced flavin adenine dinucleotide; CoA—Coenzyme A; NAD^+^/NADH—oxidized/reduced nicotinamide adenine dinucleotide; PLP—pyridoxal phosphate; THF—tetrahydrofolic acid). (**B**) Metabolic roles of the hLADH-bearing multienzyme complexes. Metabolic steps are indicated by arrows (double-headed arrows indicate multi-step transformations, whereas bidirectional arrows indicate reversible reactions).

Dihydrolipoamide dehydrogenase (LADH, E3) is a mitochondrial flavoenzyme operating as one of the components of the α-keto acid dehydrogenase multienzyme complexes (the pyruvate dehydrogenase (PDH), the α-ketoglutarate dehydrogenase (KGDH), the α-ketoadipate dehydrogenase (KADH), and the branched chain α-keto acid dehydrogenase (BCKDH) complexes (c)) and the glycine cleavage system [1,2,3,4]. The role of LADH is to regenerate the catalytic activity of the above complexes by oxidizing their E2-subunit-bound lipoic acid (LA) prosthetic group and simultaneously reducing an NAD^+^ co-substrate (Figure 1) [5].

**Figure 2:**
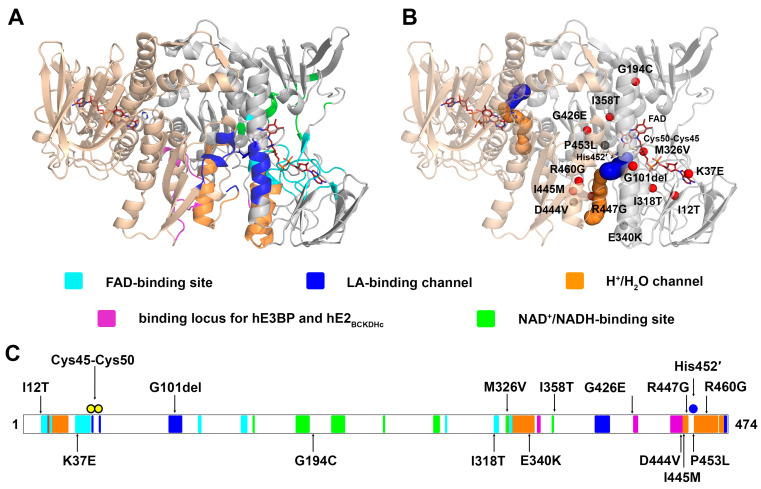
Functional regions and mutation sites in hLADH. (**A**) Functional regions are highlighted by different colors in one of the two chains in the obligate hLADH homodimer (PDB ID: 6I4Q). Parts of the monomers with no assigned functional roles are colored beige and grey. (**B**) Clinically reported disease-causing mutation sites are indicated by spheres in a single monomer; red spheres indicate variants studied in this manuscript, while grey spheres indicate three additional mutants. Solvent-accessible protein channels, computed by the Caver 3.0.1 plugin software within PyMol, are represented by colored spheres. The redox-active disulfide Cys45–Cys50, the catalytic base His452′ and the prosthetic group FAD are all represented as sticks, such as those in panel (**A**). (**C**) Sequential localizations of the functional regions, the mutation sites, Cys45–Cys50, and His452′.

The LADH reaction is governed by the ping-pong bi–bi mechanism [6], which is structurally supported by two separate substrate-binding sites; the dihydrolipoamide substrate is oxidized on the *si* face, while the NAD^+^ co-substrate is reduced on the *re* face of the isoalloxazine ring of the FAD prosthetic group [7,8]. The LADH/E3 component is a functional (obligate) homodimer since residues from both of the monomers have catalytic roles in each active site in this dimeric enzyme (Figure 2A); the redox-active disulfide bond (Cys45–Cys50; the numbering reflects the sequence of the mature human (h) enzyme) resides in one of the monomers, while the catalytic base His452′ is part of the adjacent monomer (indicated by ′) [7,8,9,10]. The dihydrolipoamide substrate can approach the active site through an approximately 10 Å long channel (Figure 2B) [11]. This channel continues towards the protein surface in a longer and narrower leg (Figure 2B) that likely provides a physical outlet for H_2_O molecules [11] and/or the catalytically generated H^+^/H_3_O^+^ [12].

Pathogenic mutations in the hLADH-coding *DLD* gene lead to LADH/E3 deficiency. Thirteen amino acid substitutions and one deletion have been described, as of today, that affect different functional regions in the hLADH, including the following: the active site or the lipoate-binding channel (G101del and P453L), the FAD-binding site (I12T and K37E), the NAD^+^/NADH-co-substrate binding site (G194C, I318T, M326V and I358T) and the dimer interface (E340K, G426E, D444V, I445M, R447G and R460G) (Figure 2B,C). These clinically relevant disease-causing variants displayed reduced availability (stability and/or tendency to complex with the other subunits) and/or impaired catalytic activity relative to the wild-type hLADH resulting in dysfunctional multienzyme complexes, which accounts for the development of severe neurological and/or cardiological symptoms, (usually episodic) life-threatening metabolic decompensation, and often premature death [13,14,15]. Selected mutants (P453L, G194C, E340K and D444V) were reported to augment the reactive oxygen species (ROS)-generating activity of hLADH [16]. ROS production was more significant under a mild acidic condition [16,17], a common clinical feature in LADH deficiency, since lactic acidosis may frequently develop as a consequence of impaired hPDHc activity.

Our laboratory initiated a profound investigation of the disease-causing variants of hLADH in 2011. We reported the specific LADH activities, in both the *forward* and *reverse* catalytic directions, and ROS-generating capacities for eight mutants (relevant to the previous paragraph). FAD content was measured for those four mutants that enhanced the ROS-generating capacity of hLADH (see again above). Oligomeric states (potential pathological monomerization) of four pathogenic mutants were studied using calibrated size-exclusion chromatography (cSEC) coupled with nanoLC-MS. Additionally, overall conformational changes were tested for eight pathogenic mutants via circular dichroism (CD) spectropolarimetry [16]. In 2016, ten clinically relevant mutants were subjected to hydrogen–deuterium exchange mass spectrometry (HDX-MS) analysis [18]; this study provided the very first pieces of direct structural information on pathogenic hLADH variants (although only at the peptide level) [19]. In 2018, the first high-resolution crystal structure was published [9], which was soon followed by six others [10]. The current study complements the previously published research data aiming again at revealing novel molecular pathomechanisms underlying hLADH deficiency. Specific catalytic activities, including ROS-generating capacity, oligomerization state and FAD loss were measured for several disease-causing mutants. Two high-resolution mutant crystal structures were also determined and analyzed.

## 2. Results

**Figure 3:**
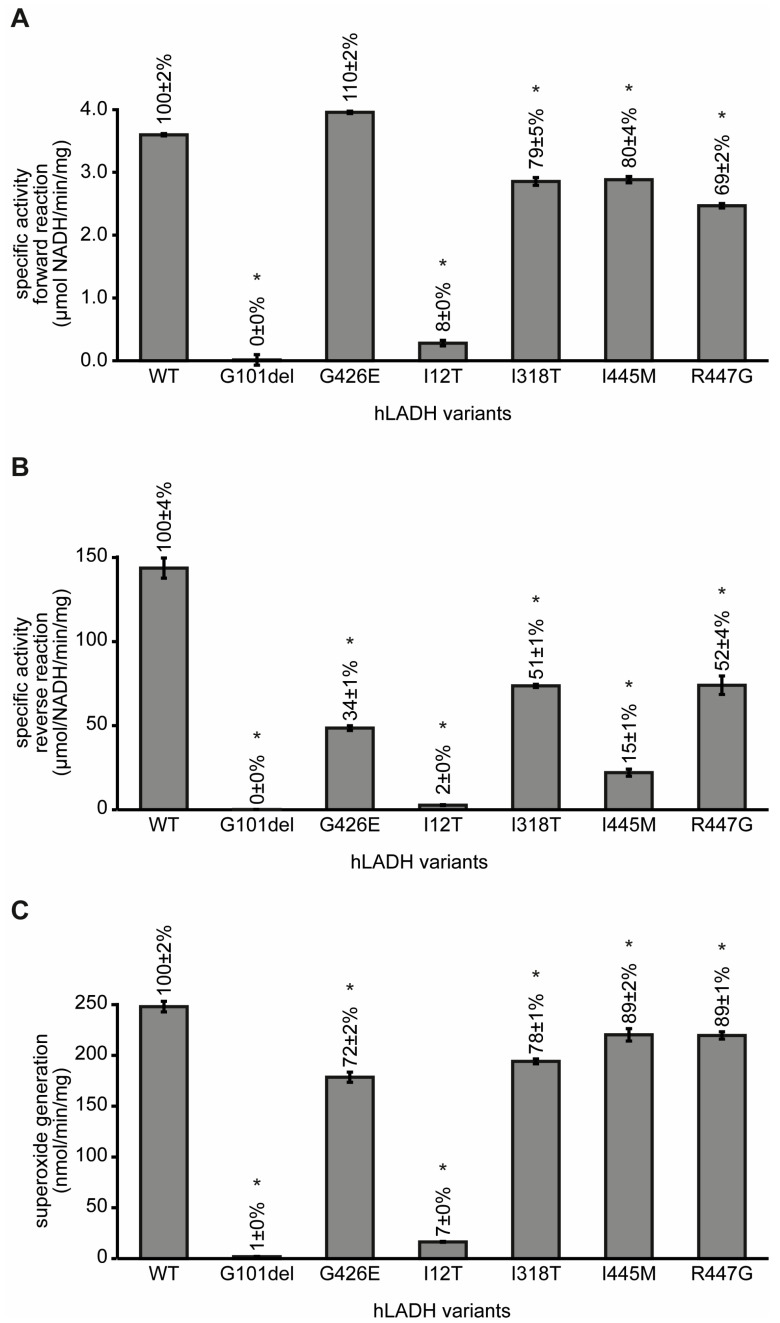
Functional loss and ROS-generating potential in selected pathogenic hLADH variants. Residual specific enzyme activities in the *forward* (**A**) and *reverse* (**B**) catalytic directions, as well as ROS-generating capacities (**C**) of selected disease-causing hLADH mutants were determined via spectrophotometry and compared to hLADH. The normal LADH reactions were followed via measuring the generation (*forward*) or consumption (*reverse*) of NADH at 340 nm. The capacity for superoxide generation was monitored in the *reverse* catalytic direction via the reduction of partially acetylated cytochrome c (ac-cyt c) at 550 nm. Reaction conditions: (**A**) 50 mM K-PO_4_ (pH 7.3), 123.45 ng of protein, 165 µM NAD^+^, and 0.9 mM dihydrolipoic acid (DHLA); (**B**) 50 mM K-PO_4_ (pH 7.3), 12.345 ng of protein, 165 µM NADH, and 0.9 mM lipoamide (LAM); (**C**) 50 mM K-PO_4_ (pH 6.3), 2.47 μg of protein, 50 μM ac-cyt c, and 165 μM NADH. Reactions were initiated by the addition of NADH (*reverse* LADH and ROS-generating activities) or DHLA (*forward* LADH activity) after incubation at 37 °C for 15 min. All of the activity measurements were carried out in two sets; G426E-, I445M-, and R447G-hLADH were measured in one round, while I12T-, G101del-, and I318T-hLADH were measured in another round. The control activities (for hLADH) in these two rounds differed by <15%; hence, they were averaged and the resultant value was applied to evaluate all the mutants. Error bars indicate the S.E.M. (standard error of the mean) with confidence intervals. The asterisk (*) denotes a statistically significant result (*p* < 0.05).

Residual catalytic activities of six clinically relevant disease-causing hLADH variants were measured (see Figure 3). The R447G, I318T, and I445M substitutions all lowered the specific LADH activities, both in the *forward* and *reverse* catalytic directions, relative to the wild type; the changes in the *reverse* activity were more pronounced (48, 49, and 85% activity losses, respectively). The G426E substitution altered only the *reverse* LADH activity (residual activity: 34%). G101del- and I12T-hLADH virtually lost their catalytic power in both catalytic directions of the LADH reaction, and also to produce ROS. None of the investigated variants stimulated the ROS-generating activity of hLADH.

**Figure 4:**
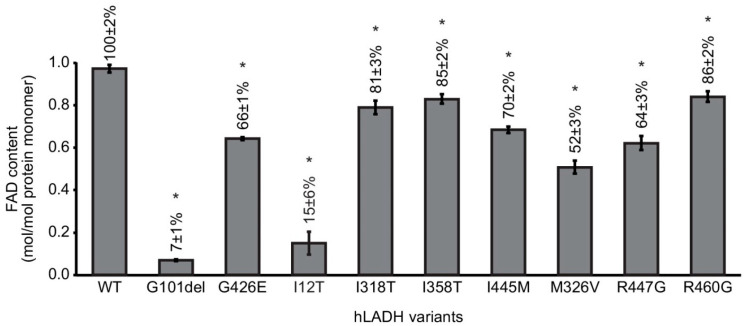
FAD loss in selected hLADH variants. FAD contents in the hLADH mutants were determined via spectrophotometry at 455 nm; FAD was first liberated from the protein-bound state via heat denaturation. FAD content is expressed relative to that of hLADH (100%: 1 mol FAD/1 mol protein monomer). Error bars indicate the S.E.M. All the differences are statistically significant (*p* < 0.05, denoted by *).

The pathogenic substitutions studied all reduced the characteristic 1 mol FAD/1 mol protein monomer FAD content of the hLADH enzyme (Figure 4). FAD content proved to be above 80% (0.8 mol FAD/1 mol hLADH monomer), compared to reference, in the I318T-, I358T-, and R460G-hLADH and 50–80% in the I445M-, G426E-, R447G-, and M326V-hLADH variants. The most significant FAD losses were induced in the G101del- and I12T-hLADH variants resulting in a 0.07 and 0.15 mol FAD/1 mol protein monomer, respectively.

**Figure 5:**
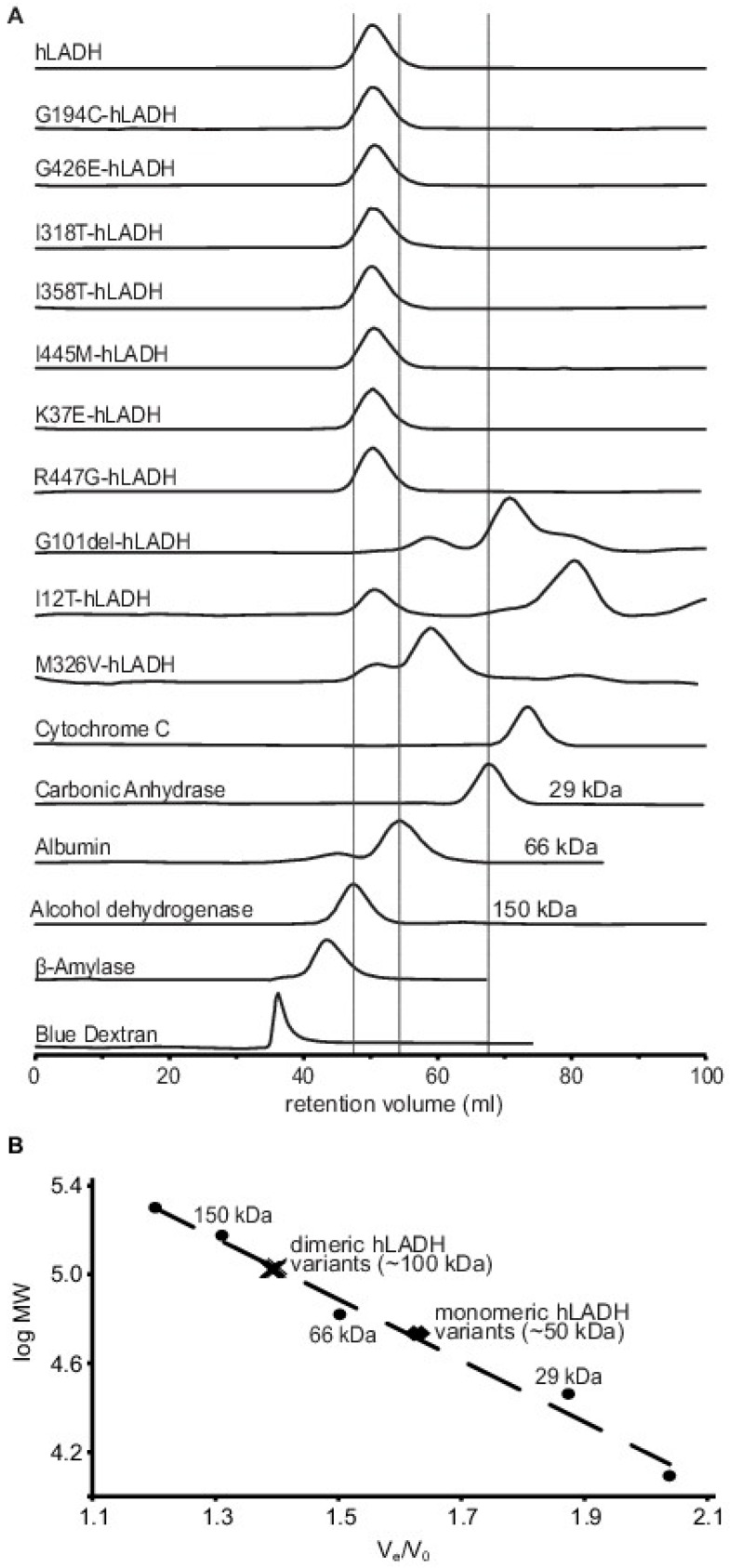
Calibrated size exclusion (FPLC) chromatography (cSEC) analysis of selected disease-causing hLADH variants. (**A**) The analytical chromatographic elution profiles of the investigated hLADH mutants, in comparison with those of hLADH and molecular weight (MW) reference proteins (from Sigma-Aldrich), represented via arbitrary peak intensities as a function of retention/elution volume (denoted as V_e_ in panel (**B**)); peak intensities in the individual chromatographic traces were normalized for better representation. The retention volume of Blue Dextran determined the void volume of the chromatography column (denoted as V_o_ in panel (**B**)). The other reference proteins assigned molecular weight ranges on the column. Single dominant chromatography peaks were present in the 66–150 kDa MW range (referenced by albumin and alcohol dehydrogenase, respectively, and illustrated by vertical straight grey lines) for most hLADH variants studied, except for I12T-, M326V- and G101del-hLADH (see text); the approximate MW of the dimeric hLADH is 100 kDa. The 29–66 kDa MW range (referenced by carbonic anhydrase and albumin, respectively) is also marked (by again another straight vertical line) to reveal potential pathogenic dissociation of the obligate hLADH homodimer (relevant here to G101del- and M326V-hLADH variants, with expected MWs of ~50 kDa). (**B**) The logMW-V_e_/V_o_ calibration curve, which is theoretically expected to be linear, could successfully be orchestrated via transforming data from panel A. Values for the applied protein references are denoted by black filled circles, with the added designation of the MW values of potential interest and crosses label the apparently dimeric hLADH variants, whereas black diamonds designate the two putatively monomeric hLADH mutants; theoretical MWs were calculated at https://web.expasy.org/compute_pi/ (accessed on 18 August 2021). Further experimental conditions are detailed in Section 4.

Monomerization of selected disease-causing hLADH variants, as a potential molecular pathomechanism in hLADH deficiency, was studied via cSEC analysis. The G194C-, G426E-, I318T-, I358T-, I445M-, K37E-, R447G- and I12T-hLADH variants displayed no monomerization (Figure 5). G101del-hLADH appears to fully dissociate into monomers, while M326V-hLADH predominantly is also in the monomeric form. G101del-, I12T- and M326V-hLADH are very low-expressing variants that often elute from the affinity column together with relatively many contaminating proteins (which were bound unspecifically to the column), unlike most other hLADH variants in our hands (see Figure S1 in [16] for reference); for these mutants, heterogeneities of the consecutive purified protein preparations were rather uncontrollable, according to SDS-PAGE analyses, while subsequent gel filtration steps proved to be preparatively superfluous and also quite diverse analytically, owing primarily to the especially low overall yields. We performed multiple cSEC analyses on these three proteins utilizing several protein preparations and Figure 5 represents the best chromatograms of all. Fractionation/isolation and MS analysis of the larger contaminating peaks for G101del- and I12T-hLADH were attempted, but failed, due likely again to the quite low yields in both cases. The most satisfying SDS-PAGE analysis results for these three proteins can be seen in Figure S1.

**Figure 6:**
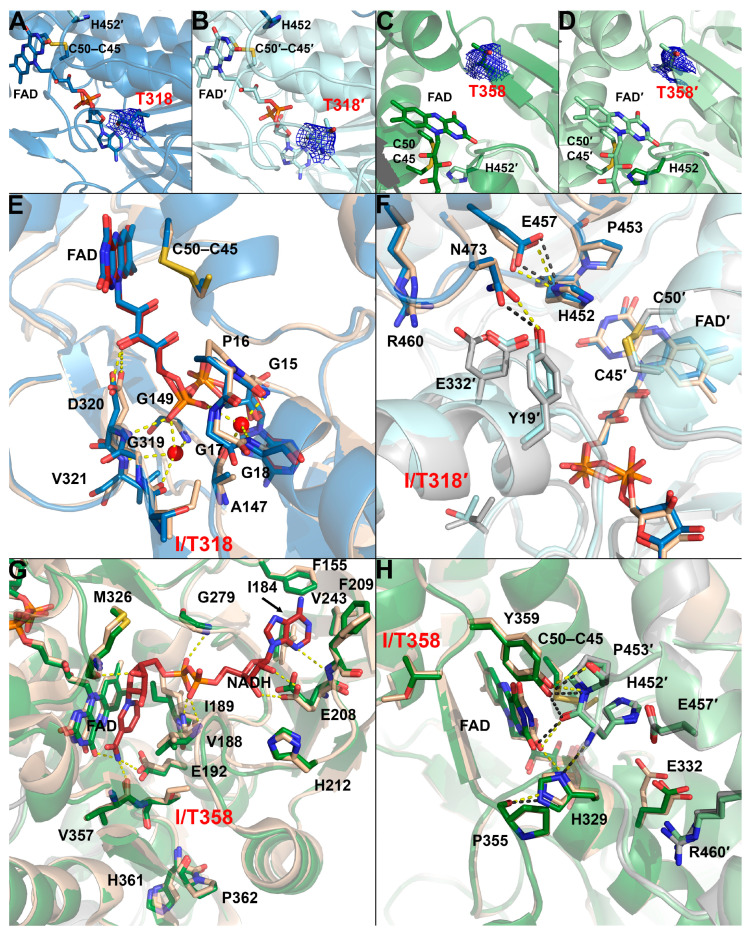
Crystal structures of the I318T- and I358T-hLADH variants. (**A**–**D**) (2mF_o_-DF_c_) Composite omit maps contoured at 1.0σ are shown as blue meshes around the substituted residues in chain A (**A**) and B (**B**) of I318T-hLADH, as well as in chain A (**C**) and B (**D**) of I358T-hLADH. The two chains in I318T- and I358T-hLADH are colored with two shades of blue and green, respectively. The FAD prosthetic group, the active site disulfide bridge (Cys45–Cys50) and the catalytic base (His452′) are all depicted as sticks. (**E**,**F**) Aligned structures of I318T-hLADH and hLADH (PDB ID: 6I4Q) are shown for part of the FAD binding site (**E**) and the active site (**F**). Structural water molecules are shown as red spheres. Selected H-bonds (see text) are displayed as dashed lines; H-bonds are not shown for the mutant structure in panel (**E**), since water molecules could not be modeled there. (**G**,**H**) Aligned structures of I358T-hLADH and hLADH (PDB ID: 1ZMD in panel (**G**), PDB ID: 6I4Q in panel (**H**)) are displayed for the NAD^+^/NADH-binding site (**G**) and the active site (**H**). The arrow points to the side chain of residue I184 behind the FAD. Monomers A and B in hLADH are beige and grey, respectively, in panels (**E**–**H**).

Seven pathogenic hLADH mutants with no high-resolution crystal structures (I318T-, I358T-, K37E-, E340K-, G101del-, I12T-, and M326V-hLADH) were subjected to crystallization trials. Structures could be determined for the I318T- and I358T-hLADH variants with 2.89 and 2.44 Å resolutions, respectively. Statistics for data collection and refinement are shown in Table 1. It is important to note that the average B-factors proved to be quite high for both of the structures. In both structures, the densities around the newly incorporated residues could not unequivocally demonstrate the presence of the threonine residues (Figure 6A–D), likely owing to the lower resolutions and high B-factors (see Table 1); however, the respective mutations were unambiguously verified via MS. In fact, the densities of the nearby FAD cofactors were also quite weak in these structures; however, relying on the R-factors, they could eventually be modeled with an occupancy of 1. When aligned with the wild-type structure, the average RMSD values for the main-chain atoms were 0.69 and 0.61 Å for I318T- and I358T-hLADH, respectively. These values are virtually identical to the coordinate errors in these mutant structures (0.66 and 0.63 Å for I318T- and I358T-hLADH, respectively); these pathogenic substitutions did not induce significant alterations in the overall hLADH structure. Fine structural alterations are analyzed in relation to changes principally in catalytic activities and disease phenotypes in Section 3.

For the R460G-, G194C- and K37E-hLADH variants the present results (minor FAD loss, no monomerization and no monomerization, respectively) provided no further insights into the proposed respective molecular pathomechanisms [10,20]; hence, these mutants are not mentioned in Section 3.

## 3. Discussion

Pathogenic mutations of hLADH, the common E3 subunit of hKGDHc, hKADHc, hPDHc, and hBCKDHc, lead to the inherited, often lethal human metabolic disease of LADH/E3 deficiency. Upon the disease-causing hLADH mutations, dysfunctions of the LADH-tethering multienzyme complexes develop via multiple independent direct mechanisms: (i) decreased provision of selected pathogenic hLADH variants due to improper folding and/or stability (leading also to premature degradation), (ii) reduced specific LADH activity, and (iii) impaired interactions amongst respective complex-forming subunits. Another, although indirect, potentially compromising mechanism is that of intensive ROS-formation by selected hLADH mutants that may also negatively (oxidatively) impact the cognate dehydrogenase complexes themselves.

The specific (*forward* and *reverse*) catalytic activities of hLADH were compromised to various degrees by the pathogenic amino acid substitutions studied (G101del, I12T, I318T, G426E, I445M, and R447G), as expected. Similarly to those of another set of disease-causing hLADH variants [16], the residual LADH activities did not correlate well with the localization of the mutation sites. The studied mutations generally induced more significant losses in the LADH activity in the *reverse* catalytic direction. Among the pathogenic hLADH mutants investigated here, there were none that could stimulate excess ROS formation compared to reference; in an earlier investigation, four pathogenic hLADH variants (D444V-, P453L-, E340K- and G194C-hLADH) exhibited enhanced ROS-forming capacities, under identical experimental conditions [16].

cSEC analysis ruled out the dissociation of the obligate hLADH homodimer as an underlying molecular pathomechanism for G194C-, G426E-, I318T-, I358T-, I445M-, K37E-, R447G- and I12T-hLADH, which is similar to the case for R460G-, D444V-, E340K- and P453L-hLADH, as determined in a previous study [16]. However, G101del- and M326V-hLADH appeared to dissociate, under the applied experimental in vitro conditions, a phenomenon that may also be viable in vivo. In fact, this molecular pathomechanism has already been proposed for mutations residing at the dimerization interface (D444V, E340K, R460G) [8,21,22] and in mild acidosis [23]; the aforementioned and other disease-causing hLADH variants (see also above) were tested earlier, by us and others, for potential pathological dissociation using various methods; however, hitherto none proved to present this phenomenon [8,9,10,16,19,24,25].

Another plausible explanation for the impaired catalytic functions is FAD loss. Hence, FAD content was determined in I358T-, R460G-, G426E-, I318T-, I445M-, R447G-, G101del-, M326V-, and I12T-hLADH, in contrast with hLADH. The results (Figure 4) revealed that FAD loss was indeed present, to various degrees, in each clinically relevant mutant studied; FAD losses, i.e., alterations (reductions) in the characteristic 1 (mol) FAD/1 (mol) protein monomer value, are expressed as residual FAD contents in percentages relative to those of hLADH in Figure 4. Localizations inside the hLADH protein of the disease-causing mutation sites did not correlate well with the magnitudes of the observed FAD losses, either. It is of potential interest that the residual values for the FAD content and the catalytic activities correlated quite well for the G101del mutation. Our laboratory [26] and others [27] reported the importance of FAD in the folding and stabilization of LADH; hence, FAD loss might as well, at least in part, account for the compromised protein stability and/or folding in relevant cases.

High-resolution crystal structures of seven clinically relevant disease-causing hLADH variants have already been published [9,10]. In this present contribution, we report the crystal structures of the pathogenic I318T- and I358T-hLADH variants at 2.89 and 2.44 Å resolutions, respectively. Below, experimental data, collected here or before by us, on residual catalytic activities including the capacity for ROS generation, FAD loss, oligomeric state, and atomic-level structure as well as clinical results reported elsewhere are cross-correlated for various pathological hLADH mutants and conclusions are drawn in terms of the respective molecular pathomechanisms. Crystallization of the G101del-, M326V-, and I12T-hLADH variants has persistently been unsuccessful in our hands, due likely to conformational instability/inhomogeneity. There are two other pathogenic hLADH mutants, E340K- and K37E-hLADH, which we possess no crystal structures for, yet; crystallization trials are still underway for these mutants. Thus, at present, atomic-level information is not yet available to structurally interpret the biochemical results for five pathogenic hLADH mutants.

Table 2 summarizes the most relevant past and present in vitro and clinical laboratory results on all the 14 pathogenic hLADH variants.

### 3.1. G101del- and M326V-hLADH

Common features of the G101del and M326V disease-causing mutations are their high instability, which is evident in vitro when heterologously expressed, as well as the almost complete loss of the specific LADH activities (*forward* and *reverse*) and ROS-generating capacity. Kim, H. et al. also reported the unstable nature of the recombinant G101del-hLADH [34] and M326V-hLADH mutants [38]; the two mutants could not be examined at all due to unsuccessful purification. Such drastic decreases in stability must manifest in vivo as well and hence contribute significantly to the respective pathomechanisms. The structural basis of the instability appears to be unequivocal only for the G101del mutation. The residue Gly101 resides in an α-helix that contributes to the formation of the entrance of the LA-binding channel in the wild-type enzyme. A single-residue deletion in the midst of this structurally important helix may indeed lead to quite significant local—and even relayed or overall—structural rearrangements and/or improper folding. These all can potentially manifest in a compromised active site and impaired FAD binding accounting for the experimentally observed virtually lost catalytic function and FAD content.

The M326V substitution virtually exterminated both the LADH and ROS-forming activities, similarly to the G101del variant. These phenomena cannot solely be explained via the observed FAD loss (0.52 mol FAD/1 mol protein monomer). According to the crystal structure of the wild-type hLADH in complex with the NADH co-substrate, the residue Met326 is involved in the stabilization of the isoalloxazine ring of the FAD prosthetic group and also in NAD^+^/NADH binding. Consequently, the substitution of this residue can evidently account for the diminished catalytic activities as well as the partial FAD loss.

Irrespective of all the above putative—but still quite plausible and potentially indeed contributing—molecular determinants, the likely most convincing results in terms of potential molecular pathomechanisms for these two disease-causing mutations are obtained via cSEC analysis. Since LADH activity demands there to be residues from both monomers in each active site in the hLADH homodimer, we propose that in the cases of G101del- and M326V-hLADH the predominant molecular pathomechanism is the dissociation of the obligate/functional homodimer.

It is quite dubious to directly correlate the in vitro experimental results with either the clinical symptoms or in situ/ex vivo measurements as neither of the two mutations presented itself in the homozygous form in any proband reported. These phenomena on the other hand might even potentially verify our data in a way that suggests that the drastic reductions in stability and/or activity by the M326V or G101del mutations would have likely been lethal prematurely or even in utero unless expressed together with a more stable and/or active variant, such as I318T (G101del/I318T [40]) or E340K; the G101del and M326V mutations both presented themselves together with the latter mutation in compound heterozygous patients (see Table 2) [28,29].

### 3.2. I12T-hLADH

The I12T substitution impaired the protein stability, similarly to the G101del and M326V mutations, but led to no dissociation of the functional homodimer. The residual LADH activities were 8 and 2% for the *forward* and *reverse* catalytic directions, respectively, which were associated with 85% FAD loss. Previously, Kim et al. reported an unaltered k_cat_ and 2.4-fold increase in K_M_ toward NAD^+^, relative to the wild type, for the recombinant I12T-hLADH mutant [50]. Ile12 is a conserved residue in the FAD-binding domain of hLADH, where it presumably participates in the orientation of the adenylyl moiety of FAD through hydrophobic interactions. No high-resolution structural information is available for the I12T-hLADH, yet; however, it can reasonably be assumed that the Ile to Thr substitution introduces novel polar contacts that may potentially perturb FAD binding.

The pathogenic I12T substitution was described in two related compound heterozygous patients; the proband with the I12T/G194C mutations had recurrent metabolic acidosis, liver dysfunction, encephalopathy, and myocardial dysfunction, whereas the cognate individual carrying the I12T and E340K substitutions displayed developmental delay and microcephaly [30]. The overall KGDHc and BCKDHc activities were both significantly reduced, while the overall PDHc activity was borderline normal for both patients when measured from fibroblasts (see Table 2) [30]; the compromised protein stability, affinity for FAD, and catalytic activities, all potentially interconnecting with one another for I12T-hLADH may well account for these overall functional losses.

### 3.3. I318T-hLADH

**Figure 7:**
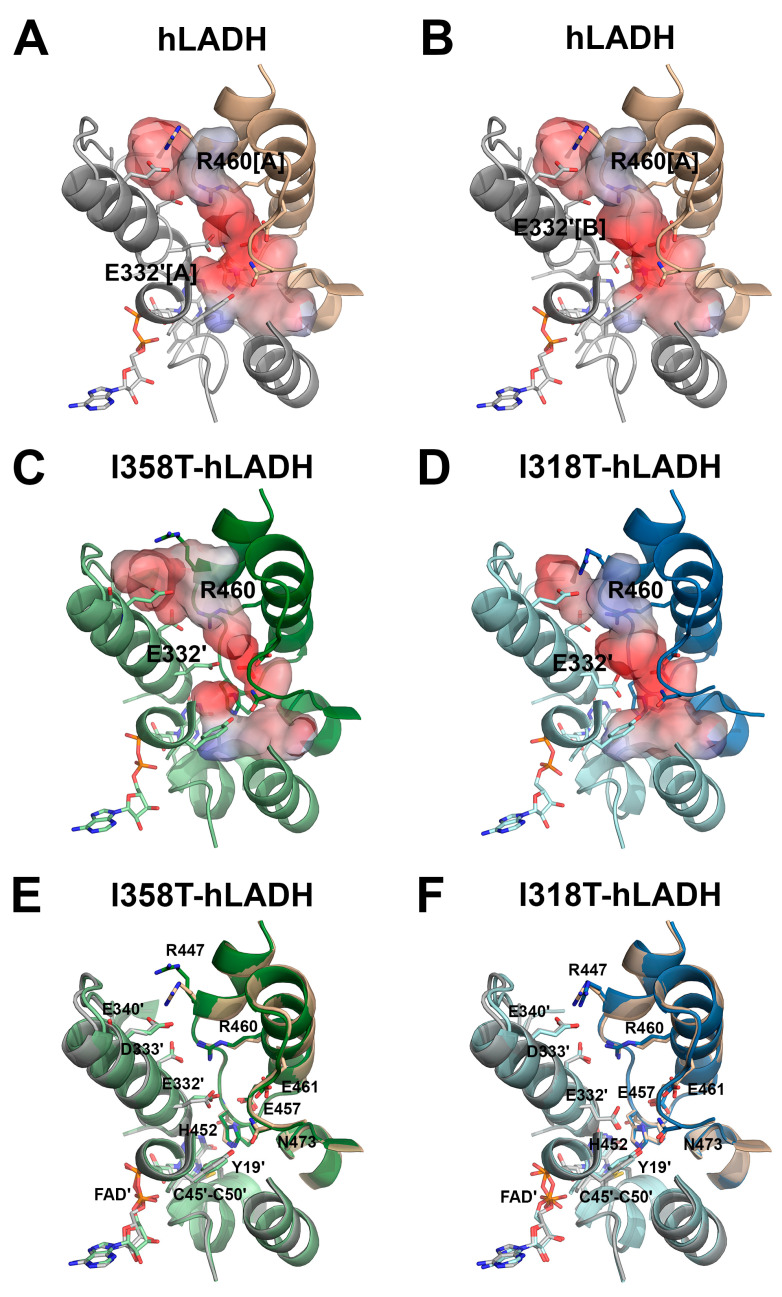
Geometry and surface potential alterations in the substrate-binding and H^+^/H_2_O channels induced by the I318T and I358T substitutions. (**A**,**B**) Channel surface potentials in hLADH for the Arg460[A]-Glu332[A] (**A**) and Arg460[A]-Glu332[B] (**B**) side chain conformations. (**C**) Channel surface potentials in I358T-hLADH. (**D**) Channel surface potentials in I318T-hLADH. In (**A**–**D**), surface potentials are displayed on an arbitrary scale (red: negative; blue: positive). The Pro16-Gly29 helix is partially elided for clarity and thus Lys24 (contributing to surface polarity) is also omitted. (**E**) The H^+^/H_2_O channels and relevant polar side chains are shown in I318T-hLADH (dark/light green; FAD′: green) and hLADH (beige/grey; FAD′: grey). (**F**) The H^+^/H_2_O channels and relevant polar side chains are shown in I318T-hLADH (dark/light green; FAD′: green) and hLADH (beige/grey; FAD′: grey).

The I318T substitution altered the specific LADH activities in both catalytic directions (79 and 51% residual activities in the *forward* and *reverse* directions, respectively) and induced less than 20% FAD loss in the recombinant enzyme, even though the residue Ile318 resides in the FAD-binding region in hLADH. This residue in hLADH is involved in the formation of a β-sheet structure that interacts with the FAD at several points (mostly at the β-turn segments) and is also proximal to the entrance of the NAD^+^/NADH-binding pocket and residues that directly participate in NAD^+^/NADH binding (Met326, among others). The residue Ile318 itself coordinates a H_2_O molecule through its carbonyl oxygen (along with Val321(N) and Gly149(N)), which in turn interacts with one of the phosphates in FAD (Figure 6E). In all the crystal structures of hLADH and its pathogenic mutants hitherto studied, two such H_2_O molecules could always be modeled on the two sides of this same phosphate; hence, those may well be conserved (Figure 6E). Thus, the residue Ile318 likely plays a role in FAD coordination in hLADH. However, the crystal structure of I318T-hLADH revealed no significant structural alterations throughout the structure that support our biochemical/functional results, at least in the *forward* (physiologically more relevant) direction. In the mutant structure, H_2_O molecules could hardly be modeled (due likely to the high B-factors), even in the highly conserved positions near the FAD; therefore, no conclusions could be drawn regarding the effects of the substitution on the above detailed H_2_O-mediated interaction with the FAD. The substitution triggered only minor displacements in the main chain of the directly affected peptide segment (Ile315-Val321) (Figure S2); therefore, the above-mentioned key interaction with a water molecule cannot completely be ruled out. In the active site, minor dislocations of the His452 and Glu457 side chains could be observed (Figure 6F). As a result, these two side chains moved apart by a fraction of an angstrom, perhaps also weakening their otherwise crucial interaction. Alternative conformations for amino acid side chains could not be modeled because of the relatively low resolution; the residues Glu332 and Arg460’ were no exceptions. The Glu332 side chain appeared to become extended towards the active site (Figure 7D,F); however, the slightly blurred density did not exclude the flexibility of the side chain, either. If we assume a dominant conformation, which extends towards the active site, then that may indeed be capable of perturbing the polarity and geometry of the active site (Figure 7D,F) and eventually LA binding. The Arg460 side chain is in a conformation that potentially permits the formation of a H^+^/H_2_O channel of a larger diameter.

The I318T substitution was described in a compound heterozygous patient also carrying the G101del mutation [40]. The patient was diagnosed with Leigh syndrome; severe symptoms—encephalopathy, liver dysfunction, lactic acidosis, and hypoglycemia with elevated levels of plasma amino acids and urine organic acids—started to develop at the age of 1 and then presented themselves in recurrent episodes. When measured from cultured fibroblasts, the overall PDHc and KGDHc activities were 80 and 107%, respectively, of the means of the respective reference ranges; i.e., both were within the normal range, whereas the overall BCKDHc activity was reduced to 24% of the control (Table 2) [40]. Based on the novel findings presented above, the substantially deteriorating effects of the G101del mutation appear to become somewhat balanced by the I318T substitution, which alone leads to a relatively small decrease in the *forward* LADH activity. The I318T substitution likely triggers the disassembly of hBCKDHc, but not of hPDHc or hKGDHc, whose structural basis could not be elucidated from the X-ray structure due to crystal contacts.

### 3.4. I358T-hLADH

The disease-causing I358T mutation was found earlier to quite significantly reduce the specific *forward* and *reverse* LADH activities as well as the ROS-generating activity of hLADH (in the *reverse* catalytic direction); the respective residual specific activities were of similar magnitudes (41, 30 and 53%, respectively) [16]. Apparently, the 15% FAD loss cannot account for the above activity losses. Earlier, an unaltered k_cat_ and a 2.5-fold higher K_M_ toward NAD^+^ were reported for the *forward* LADH reaction in the case of this recombinant variant, in contrast to hLADH [42].

According to the domain structure of hLADH, the residue Ile358 belongs to the dimer interface domain. However, in terms of functional pertinence, the residue Ile358 resides in the NAD^+^/NADH-binding pocket, adjacent to residues such as Val357 (Figure 6G), which directly interacts with the nicotinamide moiety of NADH (and likely also that of NAD^+^, according to a hLADH crystal structure (PBD ID: 1ZMD) published earlier [8]). The residue Ile358 is also in the close spatial vicinity of the isoalloxazine ring of the FAD prosthetic group. According to the I358T-hLADH crystal structure, the Ile358 to Thr358 substitution led to no significant structural alterations in the hLADH structure (Figure 6G,H and Figure S2). The Thr side chain occupies a smaller space compared to that occupied by Ile; therefore, the appearance of any novel steric conflict near the mutation site was highly unlikely and indeed unobserved. Upon superimposition with the hLADH crystal structure, it was also apparent that the protein backbone suffered no significant structural alterations from the mutation. Neither any considerable displacement in the neighboring peptide Cys449′-His452′, nor any significant changes in the active site could be observed (Figure S2). Near the mutation site, the dislocation of the His329 and Tyr359 side chains could be observed; the hydroxyl group of the residue Tyr359 moved closer to the carbonyl oxygen of the residue His452 (catalytic base). In the wild-type structure, the His329 side chain is in H-bonding distance to Pro355(O), His-452′(N), and FAD(O2) (Figure 6H and Tables S1 and S2; for Table S2, see also Figure S3). In I358T-hLADH, the His329(ND1)-FAD(O2) distance increased to ~3.6 Å; the weaker interaction might have affected the binding affinity and/or redox potential of the FAD, potentially contributing to the observed reduced enzyme activities. In the H^+^/H_2_O channel, the residue Glu332 could be modeled in a conformation that pointed away from the active site (Figure 7C). The slightly blurred density may again only represent side chain flexibility; however, if we assume a dominant conformation, then this can potentially affect the polarity and/or geometry of the active site and eventually substrate (LA) binding. Owing to additional minor dislocations of the channel-forming polar side chains, altered polarity and geometry could indeed be observed near the active site (see Figure 7A,C,E).

The I358T mutation has been found in a compound heterozygous patient, where the mutation in the other allele affected splicing and resulted in unstable and eventually untranslated mRNA [41]. The patient developed severe neurological symptoms, such as developmental delay, hypotonia, ataxia, microcephaly, stroke-like episodes, and tetraspasticity. The clinical laboratory tests showed elevated levels of plasma amino acids (branched-chain amino acids, glutamine and alloisoleucine), urine organic acids (lactate, branched-chain oxoacids, 2-hydroxyglutaric acid) and ketones. The enzymatic assays revealed drastic reductions in the PDHc and LADH activities when measured from muscle homogenates (14 and 29% of the control, respectively), whereas the PDHc activity was in the normal range when cultured skin fibroblasts were examined (Table 2) [41]. The reduction solely in LADH activity cannot clearly explain these results; hence, the assemblies of the respective complexes are likely also compromised.

### 3.5. G426E-hLADH

The G426E substitution affected only the *reverse* LADH reaction (34% residual-specific activity relative to the wild type). The FAD content proved to be 0.66 mol/1 mol protein monomer. Similarly to the residue Ile358, the residue Gly426 also resides in the dimerization domain near the nicotinamide-binding pocket in hLADH. According to the G426E-hLADH crystal structure [10], the G426E substitution altered the local charge distribution and induced side-chain dynamics near the nicotinamide binding site. These fine structural changes, likely reducing the affinity for NADH, may account for the compromised LADH activity in the *reverse* catalytic direction and the moderate FAD loss, even though the presence of the FAD prosthetic group in the crystal structure was indisputable.

The G426E mutation was described in a compound heterozygous patient (Table 2), a young adult (a 19-year-old man) presenting with myopathic features [43]; the other mutation assisted in the synthesis of a truncated protein. The novel results of the relevant enzyme activities above, including the virtually unaltered *forward* LADH activity in particular, are in accord with the relatively mild clinical symptoms.

### 3.6. I445M- and R447G-hLADH

The R447G and I445M substitutions lowered the *forward* LADH activity to similar degrees (residual activities: 69 and 80% of the control, respectively); the FAD losses correlated well with the respective *forward* LADH activity losses (residual FAD contents: 64 and 70% of the wild type, respectively). The magnitudes of the changes in the *reverse* LADH activity were more significant and rather dissimilar for the two mutants (residual activities: 52 and 15%, respectively, relative to the wild type).

The two mutations both reside at the homodimer interface. In hLADH, the residue Arg447 forms an inter-monomeric salt bridge with the residue Glu340′ near the H^+^/H_2_O channel exit, while the residue Ile445 resides in a H^+^/H_2_O-channel-forming α-helix with its side chain protruding into a hydrophobic environment. The relevant mutant crystal structures were determined earlier and they revealed modest structural changes in the H^+^/H_2_O channel [10]. The R447G mutation led to the loss of the above-mentioned inter-monomeric stabilizing salt bridge and a wider H^+^/H_2_O channel exit. Unlike in the hLADH structure, the Glu332 side chain could adopt only a single conformation in the I445M-hLADH crystal structure. The two substitutions led to no displacement in any catalytic or FAD/NAD^+^/NADH-binding residue. No FAD loss could be detected in these crystal structures, either.

These two substitutions are similar to the D444V and R460G mutations in affecting the dimerization surface, disrupting inter-monomeric salt bridges, and perturbing the H^+^/H_2_O channel [10]. The D444V and R460G mutations resulted in very significant activity losses [16]; however, the revealed structural changes were similar to those found in the R447G and I445M structures; overall, the observed structural alterations could not account for the activity losses. The significant LADH activity losses in all the above four variants and the enhanced ROS-producing capacity of D444V-hLADH [16] imply the potential presence of auxiliary molecular pathomechanisms, such as perturbation of the pK_a_ of the catalytic base and/or the redox potential of the FAD prosthetic group, mediated perhaps by local dynamics and/or dipole moment alterations in selected nearby α-helices.

Despite the comparable magnitudes of the activity losses in the physiologically more relevant *forward* catalytic direction, FAD losses and structural changes, the I445M and R447G mutations manifested in distinct clinical phenotypes. The I445M substitution was reported in a homozygous patient presenting with myopathic features [44]. The proband reached adulthood and the tolerable clinical symptoms only appeared episodically. The R447G mutation, however, proved to be prematurely lethal [45]. The R447G-hLADH mutant was associated with significant reductions in the overall PDHc and BCKDHc activities (residual activities: 63 and 56%, respectively) [45], whereas the overall PDHc activity was 97% of the lower value in the reference range for the I445M-hLADH mutant [44] when measured from patient fibroblasts (Table 2). Changes in dynamics induced by these mutations, presumably more pronounced for the R447G-hLADH, may also affect the association of the respective cognate multienzyme complexes.

In conclusion, clinically relevant disease-causing variants of hLADH were analyzed using various biochemical approaches for oligomerization status, the loss of the prosthetic group FAD, residual catalytic activities, ROS generation capacity, and/or high-resolution crystal structure. The novel in vitro functional and/or structural findings reported here complement earlier in vitro biochemical and clinical laboratory data on the pathogenic variants G101del-, R460G-, G194C-, K37E-, M326V-, I12T- G426E-, I445M-, R447G-, I318T-, and I358T-hLADH and hence also contribute to a more thorough understanding of the respective molecular pathomechanisms, and potentially the future pharmacological regulation, of the often prematurely lethal human disease of hLADH deficiency.

## 4. Materials and Methods

### 4.1. Chemicals

Chemicals were all purchased from Sigma-Aldrich (St. Louis, MO, USA), if not stated otherwise.

### 4.2. Protein Expression, Purification and Mutagenesis

The vector plasmid with the coding sequence of hLADH was purchased from ATUM, Inc. (Menlo Park, CA, USA). Mutations were introduced by using the QuikChange II mutagenesis kit (Stratagene; Cedar Creek, TX, USA). Subsequent plasmid isolation was carried out with the QIAGEN Plasmid Mini and Midi kits (Qiagen, Hilden, Germany). Wild-type hLADH and its pathogenic variants were expressed by applying a BL21(*DE3*)/pET-52b(+) system and purified to homogeneity utilizing a previously optimized Strep-tag-based affinity chromatography protocol in a single step [19,51]. To increase the yield, the poorly expressing I12T-, G101del-, and M326V-hLADH variants were also expressed with an N-terminal Twin-Strep-tag. Each preparation was inspected via mass spectrometry to verify the protein sequences (see a representative example in Figure S4).

### 4.3. Calibrated Size-Exclusion Chromatography

Gel filtration studies were performed at 4 °C using a HiPrep 16/60 Sephacryl S-200 HR chromatography column connected to an AKTA Purifier 10 UPC FPLC system, as described previously [16,52]. In the courses of the individual chromatography runs, various amounts of the mutant proteins were administered onto the column (40–780 μg; depending on the overall yields and final concentrations of our protein solutions). The moving phase was 150 mM ammonium acetate, the pH was 6.80, and a flow rate of 0.5 mL/min was applied. The calibration of the column was performed also via administering reference proteins with known molecular weights (MW kit from Sigma) in, again, individual runs.

### 4.4. Determination of Protein Concentration and FAD Content

Protein concentrations were determined according to the Bradford method via spectrophotometry at 595 nm [53].

FAD concentration/loss was determined, as before [16], through spectrophotometry at 455 nm using a calibration curve (1–40 μM) after FAD was liberated, from its non-covalent interactions with the hLADH variants, via 5 min boiling (heat denaturation) and 5 min centrifugation at 4 °C and 15,000× *g*.

### 4.5. Enzyme Activity Measurements

Specific enzymatic activities for both the *forward* and *reverse* catalytic directions of the physiological LADH reaction and the superoxide-generating activities were determined under the same experimental conditions as before [16] to record comparable results. All measurements were carried out at 37 °C, in 96-well EIA/RIA flat-bottom, polystyrol plates (Corning, Corning, NY, USA), using a SpectraMax 190 plate reader (Molecular Devices, San José, CA, USA).

*Forward* and *reverse* LADH activities were monitored by measuring the production and consumption, respectively, of NADH via spectrophotometry at 340 nm. Experiments were carried out using a 300 μL final reaction volume in 50 mM K-PO_4_, at a pH of 7.3, in the presence of either 165 μM NAD^+^, 0.9 mM dihyrolipoic acid (DHLA) and 123 ng of protein (*forward* direction), or 165 μM NADH, 0.9 mM lipoamide (LAM) and 12.3 ng of protein (*reverse* direction). Reactions were initiated via the addition of DHLA (*forward* direction) or NADH (*reverse* direction) after 15 min incubation at 37 °C. The molar extinction coefficients (ε) at 340 nm of the different NADH preparations (batches) used were 5512 and 5010 M^−1^cm^−1^, under our experimental conditions (the theoretical/literature value is 6220 M^−1^cm^−1^).

Superoxide generation was detected using spectrophotometry at 550 nm via the reduction of partially acetylated cytochrome c (ac-cyt c). Reaction mixtures in a final volume of 200 μL contained 50 μM ac-cyt c, 165 μM NADH, and 2.47 μg of protein in 50 mM K-PO_4_, with a pH of 6.3. Reactions were initiated by the addition of NADH after 15 min incubation at 37 °C. For the fully dithionite-reduced ac-cyt c, ε^nm^ = 14,565 or 14,378 M^−1^cm^−1^, in the different batches used, under our experimental conditions. 100 U (in 200 μL) superoxide dismutase (SOD; from bovine erythrocytes) entirely eliminated the ac-cyt c signal verifying the formation of superoxide by hLADH and its variants in this assay.

### 4.6. X-ray Crystallography and Structure Analysis

Proteins were crystallized utilizing the sitting-drop technique; 1 μL of the protein sample was mixed with an equal volume of the crystallization solution in a droplet, which was equilibrated against a 500 μL reservoir solution. All reagents and accessories for the crystallization were purchased from Hampton Research (Aliso Viejo, CA, USA). For the I318T-hLADH crystals, an optimal crystallization solution consisting of 0.2 M MgCl_2_, 29 *w*/*v*% PEG 3350 and 0.1 M Bis-Tris (pH 6.7) was used, after thorough screening. Well-diffracting I358T-hLADH crystals could not be grown under similar conditions, hence various additives were tested. Ethyl acetate in a 0.5 *v*/*v*% final drop concentration proved to provide the best crystals; 0.2 μL of 5 *v*/*v*% ethyl acetate stock solution was added to the drop after 1 μL of the protein solution was mixed with 0.8 μL of the reservoir solution [0.2 M MgCl_2_, 0.1 M Bis-Tris (pH 6.7) and 25 *w*/*v*% PEG 3350]. Crystals were cryo-protected upon “fishing” and before flash-freezing in liquid nitrogen; I318T-hLADH crystals were submerged into the crystallization solution supplemented with ethylene glycol in two (increasing) concentrations (15 and then 30 *v*/*v*%), while for the I358T-hLADH crystals, 100% Paratone N was used.

Diffraction data were collected on the BL14.1 beamline at the BESSY II electron storage ring operated by Helmholtz-Zentrum Berlin (HZB, Berlin-Adlershof, Germany) [54,55] and processed in parallel with data collection using the program XDSAPP 2.0 [56]. Both variants crystallized in the C 1 2 1 space group. Phases were obtained via molecular replacement using chain A of the wild-type hLADH structure (PDB ID: 6I4Q) in Molrep [57] (v.11.7.03) from the CCP4 suite [58]. Two monomers forming the functional homodimer were found in the asymmetric unit in both cases. Refinement was carried out using Refmac5 [59] (v.5.8.0158) and phenix.refine in Phenix [60] (v.1.14); the former program was used for rigid body refinement and the initial cycles of restrained refinement, while the latter program was applied in the final cycles of restrained refinement. Despite being a default setting in phenix.refine, real-space refinement was not applied as it resulted in higher R-factors. The models were validated using Molprobity [61] (v.4.02b-467). The atomic coordinates of the I318T- and I358T-hLADH model structures have been deposited in Protein Data Bank and are now available under the accession numbers 7ZYT and 7PSC, respectively.

The novel structures of the pathogenic hLADH variants were compared against the wild-type hLADH structure that was published by our laboratory earlier (PDB ID: 6I4Q) [10]; the mutant structures were least-squares fitted to the wild-type structure using ProFit (from http://www.bioinf.org.uk/sotware/profit accessed on 11 November 2021) to obtain overall and residue-level root-mean-square deviation (RMSD) values for the numerical characterization of the structural differences. Interactions that contributed to the stabilization of the homodimer and the FAD prosthetic group were analyzed using the program CONTACT in CCP4. Structures and surface potentials were visualized using PyMol v.1.8.4.2. (PyMOL Molecular Graphics System, Schrödinger, LLC, New York, NY, USA).

### 4.7. Statistical Evaluation of Data

Statistical differences were evaluated using the two-tailed Student’s *t*-test assuming unequal variances at *p* < 0.05.

## Figures and Tables

**Table 1:** Data collection, processing and refinement statistics *.

Structure	I358T-hLADH	I318T-hLADH
PDB ID	7PSC	7ZYT
***Data collection and processing***		
**Wavelength (Å)**	0.9184	0.9184
**Resolution range (Å)**	49.12–2.44 (2.58–2.44)	47.64–2.88 (3.05–2.88)
**Space group**	C 1 2 1	C 1 2 1
**Unit cell parameters** **a, b, c (Å)**	188.96, 61.25, 85,01	188.65, 59.00, 83.23
** 8.65, 59.00**	90.0, 101.06, 90.0	90.0, 101.4, 90.0
**Total reflections**	240,758 (39,200)	131,248 (18,568)
**Unique reflections**	35,480 (5611)	20,384 (3006)
**Completeness (%)**	98.5 (97.2)	98.3 (91.1)
**I/0.3**	13.30 (0.50)	8.37 (0.57)
**Wilson B-factor (Å^2^)**	84.89	87.28
**CC_1/2_ (%)**	100.0 (36.2)	99.8 (35.4)
***Refinement***		
**Reflections used in refinement**	35,451	20,301
**Reflections used for R-free**	1768	1010
**R-work**	0.2598	0.2504
**R-free**	0.2867	0.2870
**No. of non-H atoms**	7198	7150
** macromolecules**	7025	7012
** ligands**	162	138
** solvent**	11	0
**RMS (bonds) Å**	0.003	0.003
**RMS (angles) (°)**	0.56	0.53
**Ramachandran-favored (%)**	98.09	95.74
**Ramachandran-allowed (%)**	1.91	4.26
**Ramachandran outliers (%)**	0.00	0.00
**Rotamer outliers (%)**	4.43	6.72
**Average B-factor**	131.87	119.32
** macromolecules**	131.95	119.34
** ligands**	129.75	118.32
** solvent**	107.83	-
***Molprobity results***		
**Clashscore**	6.16 (99th percentile)	5.08 (100th percentile)
**Molprobity score**	1.83 (97th percentile)	2.18 (98th percentile)

* Statistics for the highest-resolution shell are shown in parentheses.

**Table 2:** The most relevant past and present in vitro and clinical laboratory results on all the 14 pathogenic hLADH variants ^a^.

Substitution	Type of Sample		Genotype in Patient ^b^	Residual Enzymatic Activity ^c^ (% of Control)					ROS Production (% of Control)	FAD Content (% of Control)	Disassembly of Homo-Dimer ^d^ (Y/N)	PDB ID	References
				LADH- Forward	LADH-Reverse	PDHc	KGDHc	BCKDHc					
**G101del**	PT	L, F, H, M, LV	G101del/E340K	9^L^, 2^F^, 4^H^, 11^M^, 10^LV^		13^L^, 12^F^, 22^H^, 14^M^, 21^LV^	6^F^, 1^H^, 1^M^, 3^LV^						[28]
	RP			**0**	**0**				**1**	**7**	**Y**		this study
**M326V**	PT	F, M, L	M326V/E340K	14^F ‡^, 6^M ‡^, 12^L ‡^		44^F ‡^, 14^M ‡^, 38^L ‡^							[16,29]
	RP			5	7				2	**52**	**Y**		[16] and this study
**I12T**	PT	F	I12T/E340K	9	28	59	25	63					[30]
		F	I12T/G194C	10	30	69	44	58					[30]
	RP			**8**	**2**				**7**	**15**	**N**		this study
**E340K**	RP			0.2–71	71–100	38			123	99	N		[16,31,32,33,34]
**G194C**	PT	M, F	G194C/G194C	28–34^F^	7–21^M^, 8–33^F^	11–12^M^, 20^F^	12–19^M^						[22,35,36,37]
		M	Y35X^#^/G194C		8–20								[35]
	RP			75–103	47–90	29	61		172	72	**N**	6I4P	[10,16,31,38,39] and this study
**I318T**	PT	F	I318T/G101del	9–29		80	107	24					[40]
	RP			**79**	**51**				**78**	**81**	**N**	**7ZYT**	this study
**I358T**	PT	M, F	I358T/ IVS9 + 1G>A		29^M ‡^	14^M ‡^, 100^F^							[41]
	RP			41–100	30				53	**85**	**N**	**7PSC**	[16,42] and this study
**G426E**	PT	F	I5Lfs*4/G426E			44	22	43					[10,43]
	RP			**110**	**34**				**72**	**66**	**N**		this study
**I445M**	PT	F	I445M/I445M	0		97 ^‡^							[44]
	RP			**80**	**15**				**89**	**70**	**N**	6I4T	[10] and this study
**R447G**	PT	F	R447G/R447G		20	63	0	56					[45]
	RP			**69**	52–92 (**52**)	28			**89**	**64**	**N**	6I4S	[10,31,32] and this study
**R460G**	PT	L, F	Y35X ^#^/R460G	1.5^L^, 14^F^		26^L^, 11^F^	20^F^						[46]
	RP			22–91	10–40	0			74	**86**	N	6I4T	[10,16,31,32,33,47] and this study
**D444V**	PT	LV	D444V/D444V		15	0	2						[22]
	RP			31	37–100	12			131	95	N	5J5Z	[9,16,21,31,32]
**P453L**	PT	F	P453L/K37E	6									[20]
	RP			9	4				230	66	N	6I4Z	[10,16]
	RP in yeast			0		0							[48]
**K37E**	RP			20–75	126				99	67–76	**N**		[16,33,49] and this study
	RP in yeast			92		88	68						[48]

^a^ Data presented in this study are highlighted in bold and colored red. While results from different groups/studies are represented as a range of values, our present data are given in brackets after the range. ^b^ Relevant only for patients’ data. For compound heterozygous patients, clinical laboratory data are shown only once in this table. ^c^ Enzymatic activities were generally measured under different assay conditions. ^d^ Although monomerization was assessed by several techniques, here only the cSEC data are represented. ^#^ The numbering reflects the immature protein sequence containing the 35 amino acid mitochondrial leader sequence. ^‡^ % of the lower limit in the control range. Abbreviations: PT = patient’s tissue; RP = recombinant protein; F = fibroblasts; M = muscle homogenate; H = heart homogenate; LV = liver homogenates; L = lymphocytes; fs = frame shift.

## Data Availability

The coordinates and corresponding structure factor amplitudes of the structures of the I318T- and I358T-hLADH variants have been deposited in the RCSB Protein Data Bank, with accession numbers 7ZYT and 7PSC, respectively. Other raw and/or processed data are available to any reader upon request.

## Supplementary Materials

The following supporting information can be downloaded at: https://www.mdpi.com/article/10.3390/ijms241310826/s1. Reference [62] is cited in Supplementary Materials.

## Abbreviations

KGDHc, alpha-ketoglutarate (also known as 2-oxoglutarate) dehydrogenase complex; KG, alpha-ketoglutarate; PDHc, pyruvate dehydrogenase complex; BCKDHc, branched-chain α-keto acid dehydrogenase complex; GCS, glycine cleavage system; KADHc; alpha-ketoadipate dehydrogenase complex; E3, (dihydro)lipoamide dehydrogenase, the common E3 subunit of the alpha-keto acid dehydrogenase complexes; LADH, (dihydro)lipoamide dehydrogenase; LA, lipoic acid; LAM, lipoamide; DHLA, dihydrolipoic acid; h, human; FAD, flavin adenine dinucleotide; NAD^+^/NADH, nicotinamide adenine dinucleotide (oxidized/reduced forms); ROS, reactive oxygen species (superoxide anion and hydrogen peroxide); wt, wild type; h, human origin; cSEC, calibrated size exclusion chromatography.
