# Supplementary material for: Structural and Biochemical Investigation of Selected Pathogenic Mutants of the Human Dihydrolipoamide Dehydrogenase
Source: Int J Mol Sci. 2023 Jun 28;24(13):10826. doi: 10.3390/ijms241310826 (PMC10341545; doi:10.3390/ijms241310826)
Supplement: Supplementary file 1 [file ijms-24-10826-s001.zip › ijms-2451683-supplementary.pdf]

Table S1. FAD-binding interactions in hLADH, I358T-hLADH and I318T-hLADH[a].

Residue (atom)	FAD atom[b]	Distance* (Å)		
		hLADH	I358T-hLADH	I318T-hLADH
Ser14(OG)	O3B	-	3.15*	-
Gly15(N)	O5B	-	-	3.51*
Pro16(N)	O2P	3.48	-	-
Pro16(N)	O1P	-	-	3.30*
Gly17(N)	O1P/2	2.95	2.69	3.12
Ile35(O)	N3A	-	3.49*	3.43*
Glu36(OE2)	N3A	-	3.55*	-
Glu36(OE1)	O3B	2.81	2.72	2.82
Glu36(OE1/2)	O2B	2.71	2.89	2.94
Glu36(OE2)	O3B	3.17	3.10	3.25
Glu36(OE1)	O4B	-	-	3.36
Lys37(N)	O2B	-	3.45*	-
Lys37(N)	N3A	3.30	3.02	3.21
Lys37(NZ)	O2B	2.93	3.06	3.17
Asn38(N)	O2B	-	3.20*	-
Asn38(ND2)	O3B	-	3.49	-
Asn38(OD1)	O2B	3.48	3.39*	2.99
Asn38(ND2)	O2B	2.99	3.07	3.01
Asn38(OD1)	O3B	-	-	3.12*
Thr44(N)	O1A	2.81	2.79	2.83
Thr44(OG1)	O2A	2.64	3.42	3.40*
Thr44(OG1)	O1A	-	-	3.07
Thr44(OG1)	O2′	-	-	3.23
Thr44(OG1)	O4′	-	-	2.87
Cys45(N)	O2′	3.38	3.45	3.44
Cys45(N)	O4′	3.26	3.29	3.47
Lys54(NZ)	N5	3.18	3.03	3.20
Lys54(NZ)	O4	2.66	2.66	3.09
Gly119(N)	N1A	2.92	3.09	2.98
Gly119(O)	N6A	2.85	3.29	2.98
Gly119(N)	N6A	-	-	3.47*
Ala147(O)	O5B	-	-	3.41*
Thr148(O)	O4B	-	3.12	-
Thr148(O)	O5B	-	3.18	-
Ser150(N)	O2A	3.35	3.45	3.39*
Asp320(N)	O1P/2	2.88	2.97	3.21
Asp320(OD1)	O3′	2.87	3.17	3.18
Asp320(OD2)	O3′	3.31	3.11	2.91
Met326(O)	O3′	3.39	2.94	3.50
Leu327(O)	O3′	-	3.52	-
Leu327(N)	O3′	3.27	-	3.51*
Ala328(N)	N1	3.34	3.48	3.38
Ala328(N)	O2	2.76	2.88	2.68
Ala328(N)	O3′	3.36	3.52	3.55*
His329(ND1)	O2	3.27	3.57	3.28
His452′(O)	N3	2.78	3.15	2.95

[a]The atomic distances were measured using the program CONTACT in the CCP4 package. The distance cut-off for the measurements was 3.6 Å; an interaction was declared to be lost in case the respective atomic distance grew greater than 3.6 Å. The displayed values are averages over the two protein monomers. Asterisks (*) mark interactions that formed in only one of the two protein monomers. The distances reported above were all manually confirmed by using the program Coot. [b]For atom designations in the prosthetic group FAD, see Figure S3.

Table S2. Intermonomeric interactions in hLADH, I358T-hLADH and I318T-hLADH[a].

	Distance (Å)		
**Residues (atoms)**	**hLADH**	**I358T-hLADH**	**I318T-hLADH**
Tyr19(OH) – Asn473′(OD1)	2.71	2.68	2.93
Tyr19(OH) – Asn473′(ND2)	-	3.50	3.49*
Gln27(NE2) – Ser471′(O)	3.07	3.40	3.25
Ile51(O) – Thr396'(OG1)	-	3.58*	3.19*
Asn58(ND2) – Arg74′(NH1)	3.23	-	3.28*
Asn58(ND2) – Asn397′(OD1)	3.24	3.25	3.33*
Asn59(OD1) – Arg74′(NH1)	3.08	3.24	3.24
Gly75(O) – Asn84′(N)	2.80	3.08	2.99
Glu77(N) – Arg82′(O)	3.00	3.04	2.96
Glu77(O) – Arg82′(N)	2.89	2.88	2.65
Glu77(OE1) – Arg82′(NE)	2.81*	-	-
Glu77(OE1) – Arg82′(NH2)	3.22*	3.37	-
Glu77(OE2) – Asn84′(ND2)	2.68	3.46*	-
Glu77(OE2) – Lys87′(NZ)	-	-	2.44*
Ser79(OG) – Ser79′(OG)	2.30*	2.81*	2.80*
Ser79(O) – Ser79'(OG)	-	-	2.55*
Ser79(O) – Ser79'(N)	-	-	2.87*
Ser79(N) – Glu80'(O)	3.60*	3.25	3.27*
Ser79(OG) – Glu80'(N)	3.56*	3.50	-
Glu80(OE1/2) – Ser79′(OG)	3.26*	3.01*	-
Gln91(NE2) – Lys395'(O)	-	-	3.56*
Gln91(NE2) – Thr396′(O)	3.15	2.94	3.24
Gln109(NE2) – Phe474′(OXT)	3.24	3.33*	-
Gln109(OE1) – Phe474' (OXT)	-	3.47*	-
Gln109(NE2) – Phe474'(O)	-	3.29	-
His329(ND1) – His452′(N)	3.05	3.04	3.10
His329(ND1) – His452'(O)	-	-	3.56*
Glu332(OE2) – His452'(ND1)	-	-	3.52*
Glu332[A](OE1/2) – Arg460′(NH2/1)	3.16[B]	-	-
Asp333(OD1) – Arg460′(NH1)	2.60[A]/2.86[B]	3.02	3.12
Asp333(OD1) – Arg460′(NH2)	3.16[A]/3.02[B]	2.78	2.98
Asp333(OD2) – Arg460′(NH1/2)	3.24[A]	3.07	3.11
Glu340(OE1) – Arg447′(NH1)	2.80	-	2.76*
Glu340(OE1) – Arg447′(NH2)	3.23	-	2.99*
Glu340(OE2) – Arg447′(NH2)	3.32	-	3.22
Glu340(OE2) – Arg447′(NH1)	3.32*	-	3.19
Tyr359(OH) – His452′ (O)	-	3.43	3.41
Tyr359(OH) – Pro453′(O)	2.90	3.15	3.01
Tyr359(OH) – Pro453′(N)	3.37	3.36	3.29
Glu363(OE2) – Arg393′(NH2)	2.80	2.88	2.87
Arg393(NH1) – Glu427′(OE2)	2.92	3.05	2.88
Glu427(OE1) – Leu455′(N)	3.29	3.20	3.26
Glu427(OE1) – Ser456′(N)	3.01	2.77	2.90
Glu427(OE2) – Leu455′(N)	2.84	2.49	2.62
Asn430(OD1) – Thr454′(OG1)	-	-	3.49*
Asn430(ND2) – Ala451'(N)	-	-	3.14
Asn430(OD1) – Ala451′(N)	2.85	2.90	-
Asn430(ND2) – Ser456′(OG)	3.04	2.76	-
Asn430(OD1) – Ser456'(OG)	-	3.42	2.73
Asn430(ND2) – Thr454'(OG1)	-	3.54*	-
Tyr438(OH) – Tyr438'(O)	-	-	3.58*
Tyr438(OH) – Asp444′(OD2)	2.84	3.25	2.76

[a]The displayed values are averages over the two (chemically identical, spatially symmetric) protein monomers. *interactions appearing only once inside a dimer (lost interaction: 3.60 Å and beyond). [A/B]alternative conformers of Arg460 (the mutants adopted conformer A)

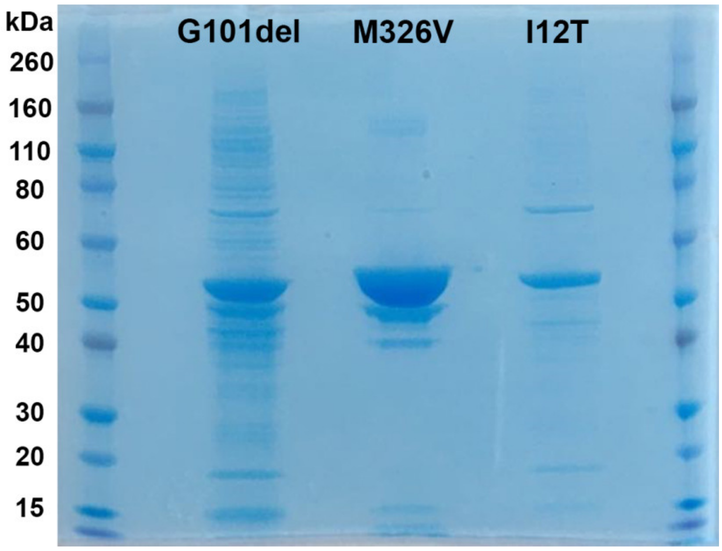

Figure S1. SDS-PAGE analysis of the G101del-, I12T- and M326V-hLADH preparations. This analysis demonstrates the integrity and purity of these low-expressing variants. It should be noted that despite having a monomeric MW of ~50 kDa, hLADH is expected to migrate at ~55 kDa in SDS-PAGE [1].

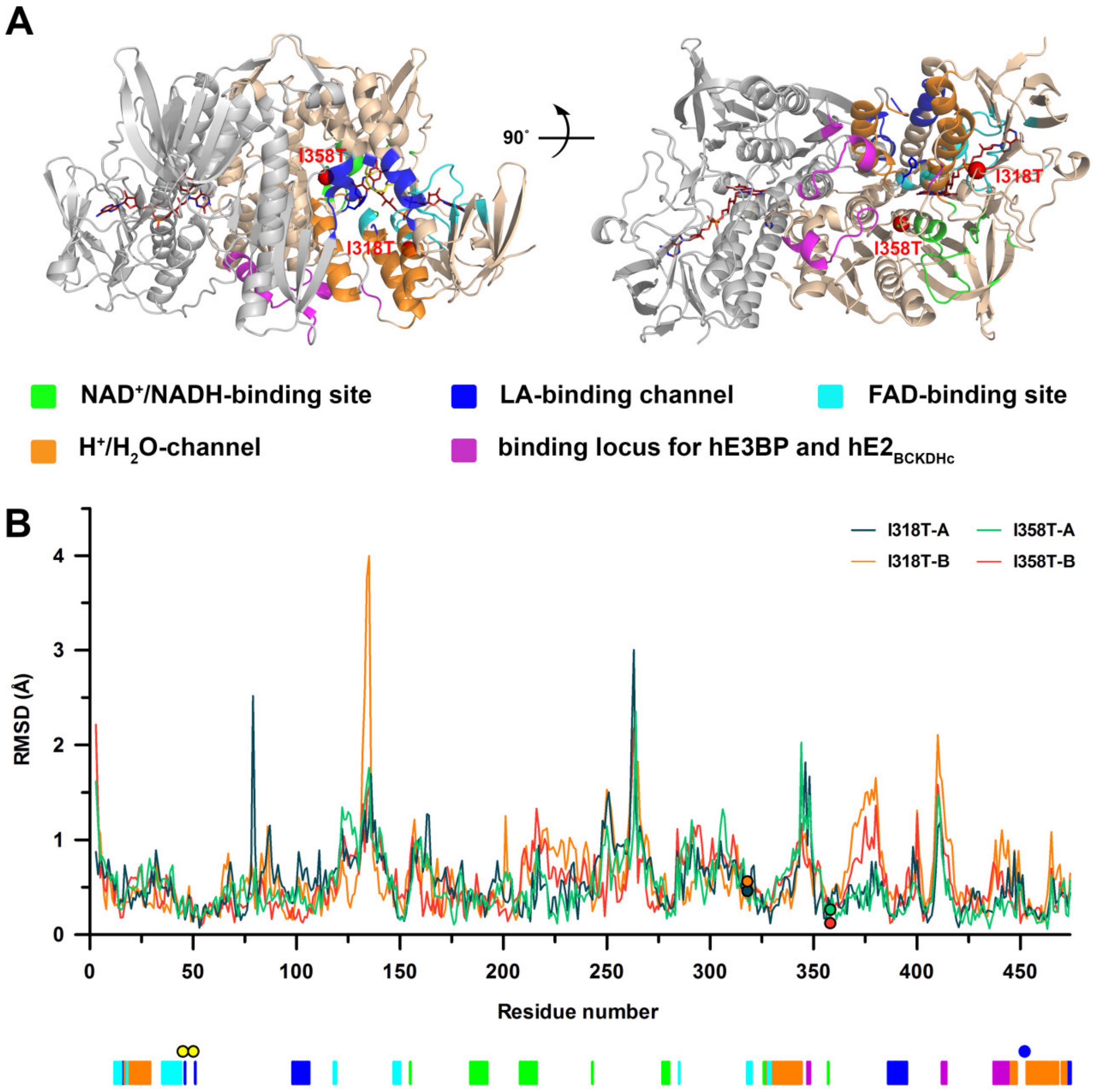

Figure S2. Functional regions in hLADH and residue displacement plots for the pathogenic I318T- and I358T-hLADH variants. (A) The functional regions are shown in two orientations in the hLADH structure (in a single copy; PDB ID: 6I4Q). The I318T and I358T substitution sites are indicated with red spheres. **(B)** Residue displacement plots for both of the monomers in the pathogenic I318T- and I358T-hLADH variants were generated by applying the main-chain atoms and the dimeric structures for fitting against the hLADH structure. Averages of the RMSD values are 0.69 and 0.61 Å for I318T- and I358T-hLADH, respectively. The RMSD values for the residues Thr318 and Thr358 are marked with circles (the applied colors are identical to the ones of the respective curves). The sequential positions of the redox-active disulfide Cys45-Cys50 and the catalytic base His452 are indicated by yellow and blue circles, respectively, under the plot. Bars below the graph indicate the sequential localizations of the functional regions shown in panel (**A**).

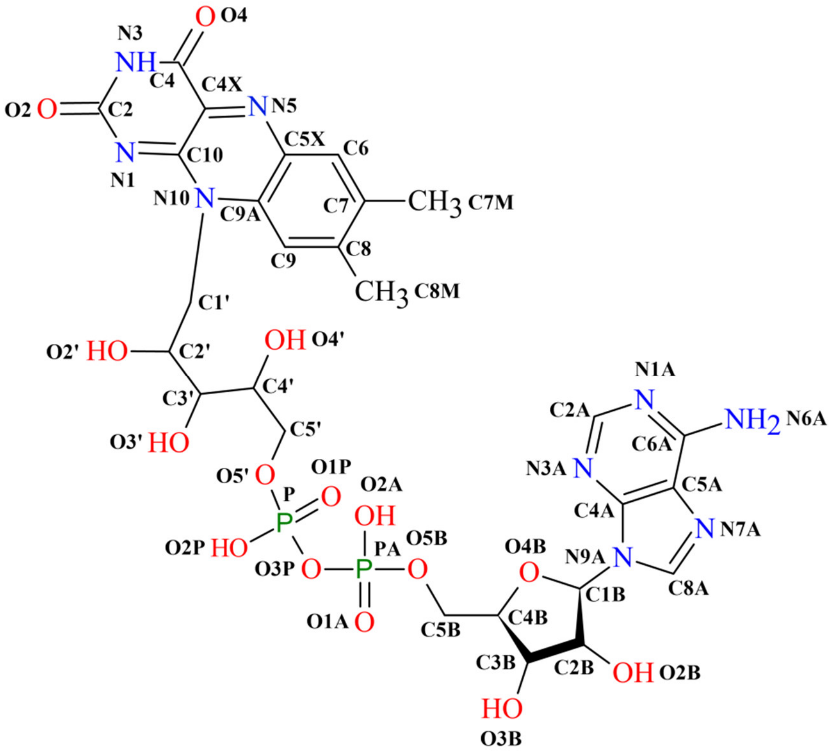

Figure S3. Atomic nomenclature of FAD in the hLADH variants. The figure was generated by using the software ChemSketch v.2022.

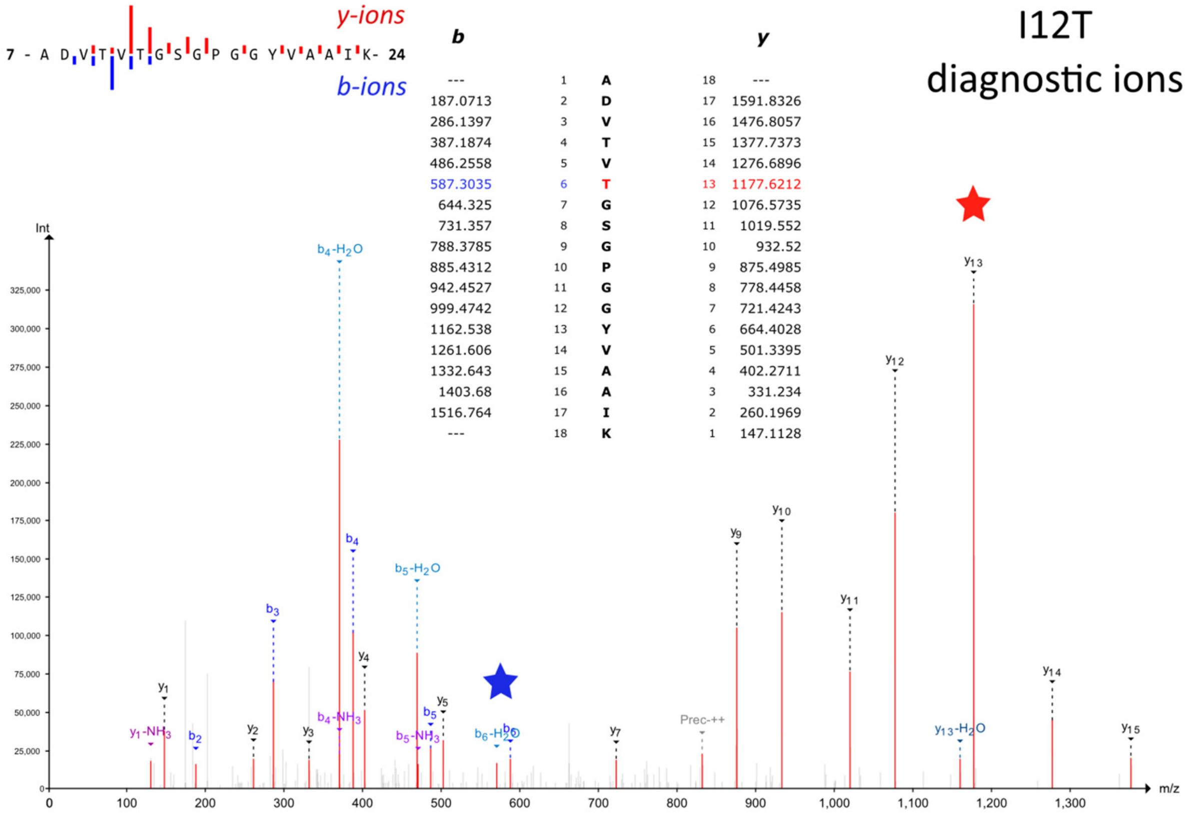

Figure S4. MS-MS spectrum of peptide 7-ADVTVTGSGPGGYVAAIK-24 in I12T-hLADH. This figure shows the ions that enable the *de-novo* sequencing of the peptide (bottom) and the mass list of the main sequence fragment ions that were expected for each amino acid (top). The diagnostic masses (in the mass table) for the respective I/T mutation are colored in blue and red for the *b*- and *y*-ions, respectively. The diagnostic b- and y-ions in the spectrum are pinpointed by a blue and red star, respectively. The *b*- and *y*-ions are fragment ions of the precursor peptide, where the peptide fragments at the peptide bond (-CO-NH-) and the charge will be localized at either the *N*- (*b*-ions) or *C*-terminus (*y*-ions). The mass difference between the consecutive ions identifies the amino acid fragment. In the figure, the main *b*- and *y*-ions are annotated with the corresponding numberings, which start from the *N*- and *C*-terminus of the peptide, respectively.

References

1. Kim, H.; Liu, T.C.; Patel, M.S. Expression of cDNA sequences encoding mature and precursor forms of human dihydrolipoamide dehydrogenase in *Escherichia coli*. Differences in kinetic mechanisms. *J. Biol. Chem.* **1991**, *266*, 9367–9373.
